# How 2 txt: an exploration of crafting public health messages in SMS

**DOI:** 10.1186/1756-0500-7-514

**Published:** 2014-08-11

**Authors:** Debra Revere, Malaika R Schwartz, Janet Baseman

**Affiliations:** Northwest Center for Public Health Practice, School of Public Health, University of Washington, 1107 NE 45th St, Suite 400, Seattle, WA 98105 USA; Department of Epidemiology, School of Public Health, University of Washington, Box 357236, Seattle, WA 98195-7236 USA

**Keywords:** Communications, Emergency preparedness and response, Health care providers, Public health, Short message service, SMS, Text messaging

## Abstract

**Background:**

Health care providers are an important target audience for public health emergency preparedness, response and recovery communications. Short Message Service or text messaging to cell phones may be a promising supplemental or alternative technique for reaching health care providers with time-sensitive public health information. However, studies to date have yet to investigate the message content and formatting requirements of providers with respect to public health alerts and advisories or sought to understand how to meet these needs using Short Message Service technology.

**Methods:**

Data collection was completed using a two-part online survey. In the first part, health care providers identified their use of different technologies for receiving information and provided input on the message components most important in a public health message. In the second part, health care providers participated in an exercise in which they shortened three public health emergency messages, ranging from 2024–2828 characters per message, to meet the 160-character limitation for text delivery. Results were analyzed to determine associations between provider types, age ranges, gender, access to various media (text, email, fax, social media, etc.), and smart phone ownership.

**Results:**

The following components were most frequently selected as essential for a public health message: Topic, Recommendation, Geographic Location, Signs & Symptoms, Population Affected, and Link to Additional Information. There was no statistically significant association between message component selection and provider type, age ranges, or gender. In the message conversion exercise, we found a statistically significant association between providers who reported receiving information by SMS and/or smart phone ownership and including a link to additional information in the converted message, ranging from 61% to over 72% on a per message analysis.

**Conclusions:**

A substantive recommendation derived from this study is that public health agencies include a link to additional website information when sending messages in SMS format. SMS could be a useful public health tool for communicating with health care providers but further investigation of how to effectively use SMS and other mobile technologies is needed to inform public health decisions regarding adoption of messaging systems utilizing these newer technologies.

## Background

Health care providers play significant roles in emergency preparedness, response and recovery; their effectiveness depends, in part, on receiving time-sensitive information from public health agencies [1]. Communication of emergency messages from public health agencies to health care providers traditionally relies on broadcast fax, telephone/land-lines, the Internet, and email. These messages are often lengthy, complicated and the number of national, state, local and professional communication channels through which they are delivered can inundate health care providers with multiple, redundant and conflicting messages [2].

Technologies such as Short Message Service (SMS) or text messaging to cell phones, twitter, and Facebook feeds may be promising supplemental or alternative techniques for reaching health care providers with time-sensitive public health information. Several studies have investigated the feasibility of using SMS for health promotion—such as chronic disease self-management for diabetes [3], dietary self-monitoring [4], and delivery of tailored health behavior interventions to reduce alcohol use [5] or smoking [6], among other programs. However, in order to use these new technologies, a traditionally composed public health emergency message must be truncated and modified. For example, Tweet text-based messages cannot exceed 140 characters in length and standard SMS is limited to 160 characters. Yet little is known about what components of a message are considered essential for conveying time-sensitive public health information to health care providers nor how to modify a lengthy public health message for delivery over SMS.

Our research objective was to identify the essential components, content and formatting of public health SMS messages to inform public health agencies regarding use of SMS for public health messaging. In this study we utilized an innovative approach, combining both passive survey questions and an interactive, participatory exercise.

## Methods

Data collection was completed through an online survey and exercise.

### Ethics

Institutional Review Board approval was sought and granted from the University of Washington (UW) Human Subjects Division (Seattle, Washington, USA).

### Recruitment and enrollment

At the conclusion of a public health messaging study [1], health care providers enrolled in the study (n = 617) were asked if they would consent to be contacted by email to participate in a future sub-study. Over 77% (n = 476) consented to be contacted and provided a current email for recruitment. The "types" of health care providers in the parent study included Advanced Registered Nurse Practitioners (ARNP), Physicians (MD), Physician Assistants (PA), Pharmacists (PHRM), and Veterinarians (VET). Providers who agreed to be contacted received an email with the sub-study description, a link to the online survey and a reply-to email address if the health care provider wanted to be removed from any future email contact regarding the sub-study. A reminder email was generated three weeks after the first invitation. Over 35% of the providers (n = 168) enrolled in this sub-study.

### Design

The survey was built using the UW's Catalyst survey tool [7] which has been approved by the UW Human Subjects Division for creating anonymous or confidential surveys. The survey consisted of two parts:

- Part 1: Essential Public Health Message Components. Providers were asked about their use of different technologies for receiving information (phone, fax, email, etc.), ownership of a device capable of receiving SMS messages and were asked to select the most important components (geographic location, source or author, contact information, etc.) to include in a public health message.
- Part 2: Message Conversion to SMS. Providers were presented with three public health emergency messages to reformat within SMS character limitations (160 characters). Messages were based on real public health communications issued to health care providers: a local/county health agency alert regarding increased reports of Norovirus-like illness (2024 characters); a state health department advisory regarding Rocky Mountain Spotted Fever (2237 characters); and a nationwide advisory disseminated by the Centers for Disease Control and Prevention (CDC) regarding a Monkeypox virus outbreak among persons who had contact with wild or exotic mammalian pets (2828 characters). Provider-constructed SMS messages were coded by two qualitative analysts, documenting inclusion of original message component in the shortened message.

Limited demographic information (provider type, gender, work setting, age range, and number of years in practice) was requested but not required for participation. Providers were not required to complete both parts of the survey.

### Statistical analysis

In addition to descriptive statistics regarding technologies through which providers received professional information and frequencies of priority message component selection in Part 1, we used Pearson's chi-squared test for independence to understand whether selection of priority message components differed for provider type, age, gender, exposure to and use of different technologies, or smart phone ownership. A p-value ≤ 0.05 indicated that differences in selection were statistically significant.

In Part 2 we examined whether converted SMS public health messages differed for provider type, age, gender, exposure to and use of different technologies, or ownership of a SMS-capable device. This analysis required a two-step variable creation: 1) An aggregate variable was created for each message component to provide a frequency measure of how often each component in the original message was included in a SMS message (see Table 1 for the list of components included in each message). Because of component variability between the original messages, this new variable was coded as being included in none, one, two, or all of the converted SMS messages. 2) To calculate whether including a message component differed by provider type or other characteristics, the aggregate variables were transformed into binary variables.

**Table 1:** **Full-length original message components and sample converted SMS messages**

	Local Health Jurisdiction	State Health Department	CDC
	Norovirus-like Illness	RMSF advisory	Monkeypox advisory
**Message components in full-length message** ^**a**^			
**Topic**	x	x	x
**Recommend**	x	x	x
**Location**	x	x	x
**Signs/Sx**	x	x	x
**Population**	x	x	x
**Link**	x	x	x
**Contact**	x		
**Report**	x	x	
**Background**		x	x
**Source**	x	x	x
**Sample SMS conversions**			
	Norovirus like illness george county. Educate pts on enteric disease. Notify institutional illness to 555-555-5555. Info at http://www.cdc.gov/norovirus/	Consider RMSF in people with abrupt onset headache, fever, rash. Do not delay treatment while waiting for serology (IgG) results. Report cases to public health.	MONKEYPOX OUTBREAK ALERT. CDC identified shipment of infected African rodents April 9th. Refer to http://ww.cdc.gov/ncidod/monkeypox/quarantineremoval.htm
	POSSIBLE NOROVIRUS IN GEORGE COUNTY. Symptoms: N/V/D; HA; lasting 24–48 hrs. Wash hands, avoid contact with infected food. Report cases to PH Dept.	1-28-11 Infected ticks transmit R. rickettsii may lead to RMSF. Diagnose sx (fever and HA), treat empirically, confirm serology, report to PH http://www.cdc.gov/rmsf	Monkeypox outbreak identified in imported rodents. Human cases confirmed. For complete data and quarantine information, see http://www.cdc.gov/incidod/monkeypox/

^a^Note that no message included the component "OtherConditions"*.*

Due to the small sample size and categorical nature of several of the variables, Fischer's exact test was used to determine if there were statistically significant differences in including components from the original public health message in the converted SMS message between provider types, age, gender, and smart phone ownership. A p-value ≤ 0.05 indicated that the differences in inclusion were statistically significant.

All analyses were conducted using the STATA Data Analysis and Statistical Software, version 12.

## Results

Of the 168 enrolled providers, 100% completed Part 1 and approximately 85% (n = 143) completed at least one SMS conversion exercise in Part 2. Table 2 details provider demographics for each survey section.

**Table 2:** **Demographics of health care providers participating in each survey section**

Provider type	ARNP	MD	PA	PHRM	VET	***Missing***
Part 1	48	54	15	31	19	1
Part 2	42	45	14	25	16	1
**Gender**		**Male**		**Female**		***Missing***
Part 1		55		111		2
Part 2		48		94		1
**Age range**	**26-35**	**36-45**	**46-55**	**56-65**	**65+ yrs**	***Missing***
Part 1	24	43	40	55	6	0
Part 2	21	40	30	47	5	0

Provider Type: ARNP = Advanced Registered Nurse Practitioner; MD = Physician; PA = Physician Assistant; PHRM = Pharmacist; VET = Veterinarian.

Nationwide adult ownership of smart phones currently stands at 56% with higher rates noted among college graduates; 19–35 year olds; and those with an annual household income of ≥ $75,000 [8]. Table 3 shows that providers' ownership of smart phones (58.3%, Part 1 n = 98; 61.5%, Part 2 n = 88) is higher than the national adult ownership rates. This may reflect the higher educational achievement and income levels of health care providers.

**Table 3:** **Provider smart phone ownership compared to national smart phone ownership rates**

	Sample part 1	Sample part 2
**Total**	98/168 (58.3%)	88/143 (61.5%)
**ARNP**	29/48 (60.4%)	26/42 (61.9%)
**MD**	35/54 (64.8%)	31/45 (68.9%)
**PA**	10/15 (66.7%)	9/14 (64.3%)
**PHRM**	17/31 (54.8%)	15/25 (60.0%)
**VET**	7/19 (36.8%)	7/16 (36.8%)
**Male**	34/55 (61.8%)	31/48 (64.6%)
**Female**	64/111 (57.7%)	57/94 (60.6%)
**26-35 years**	15/24 (62.5%)	14/21 (66.7%)
**36-45 years**	27/43 (62.8%)	25/40 (62.5%)
**46-55 years**	27/40 (67.5%)	21/30 (70.0%)
**56-65 years**	27/55 (49.1%)	26/47 (55.3%)
**66+ years**	2/6 (33.3%)	2/5 (40.0%)

### Survey part 1: prioritization of essential public health message components

Providers were asked to identify the different technologies through which they receive professional information and the five components most important to include in a public health message.

#### Part 1 communication technologies exposure

**Figure 1:**
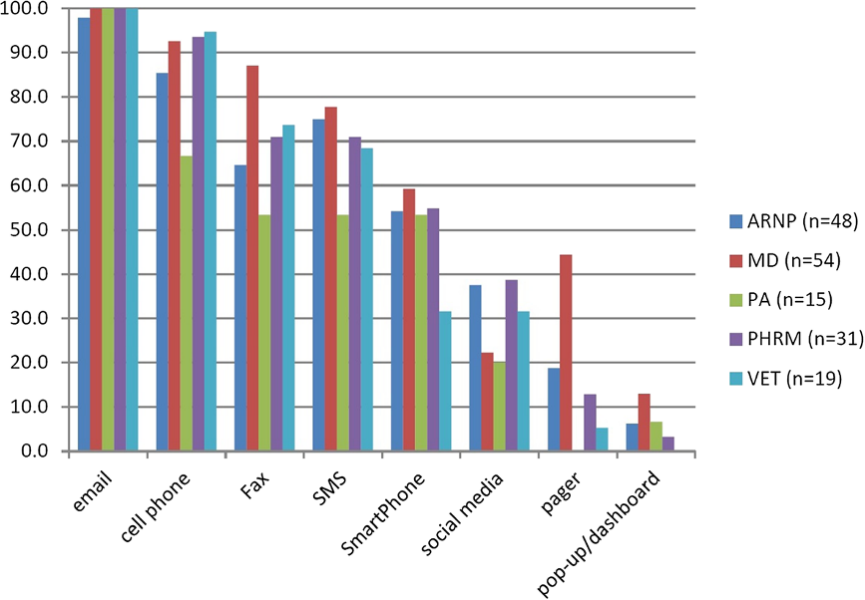
**Technologies through which Part 1 health care providers receive professional information.**

Figure 1 illustrates the range of technologies through which providers could receive professional information in descending order of frequency: email, cell phones, fax, SMS, and smart phones (defined as iPhones or Blackberries). Fewer than 50% of any provider type reported using "social media" (defined as Facebook, Twitter, etc.), pagers, browser pop-ups or dashboards to receive professional information.

#### Exercise: identification of priority public health message components

**Figure 2:**
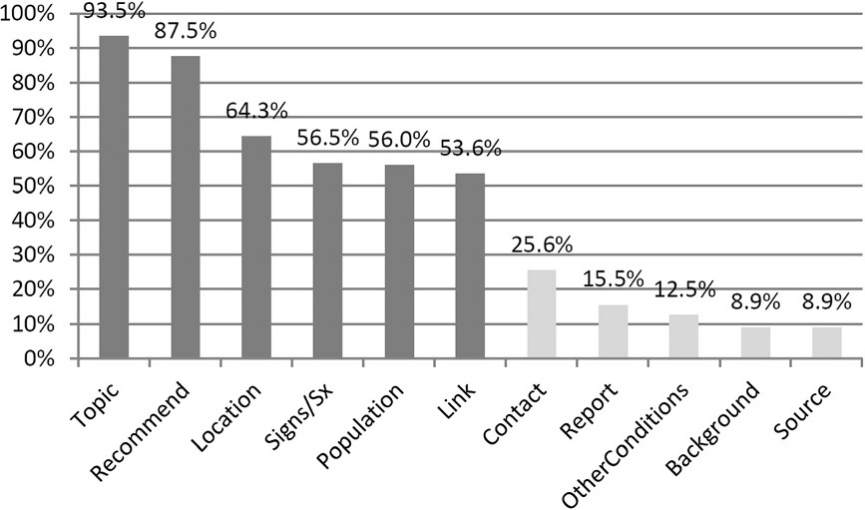
**Frequencies of public health message component selection.**

Table 4 details the list of components for prioritization. The most frequently selected components, as seen in Figure 2, were Topic, Recommend, Location, Signs/Sx, Population and Link. Pearson's chi-squared test for independence was used to determine if selection of priority message components differed significantly by provider type, age, gender, or smart phone ownership. Selection of "Recommendation" was statistically significant for age (p = 0.009) and smart phone ownership (p = 0.025). Selection of "OtherConditions" was statistically significant for smart phone ownership (p = 0.007). Provider type and gender were not found to be independently associated with component prioritization at a significance level of 0.05.

**Table 4:** **List of public health message components for prioritization in part 1**

Component	Definition
Topic	Alert or advisory topic or event
Background	Background or history of the Topic
OtherConditions	Other conditions related to the topic or event (e.g., air quality advisory impacting asthma patients)
Location	Geographic location affected by the Topic
Link	Link to more information, e.g., a link to a web page or supporting document
Population	Population (e.g., age group) affected
Contact	Public Health Department contact information, e.g., phone or fax number
Report	Instructions for reporting an incident of the Topic to the Public Health Department
Recommend	Suggested responses, requested actions or treatment instructions
Signs/Sx	Signs and symptoms regarding the Topic
Source	Source of the advisory or alert, e.g., CDC, Department of Health, etc.

### Survey part 2: message conversion to SMS

The second part of the survey asked providers to convert a standard public health message into a 160 character (i.e., suitable for SMS delivery) message in a SMS conversion exercise. Of the original sample, 143 providers completed one (n = 4; 2.8%), two (n = 5; 3.5%), or three (n = 134; 93.7%) message conversions.

#### Part 2 communication technologies exposure

**Figure 3:**
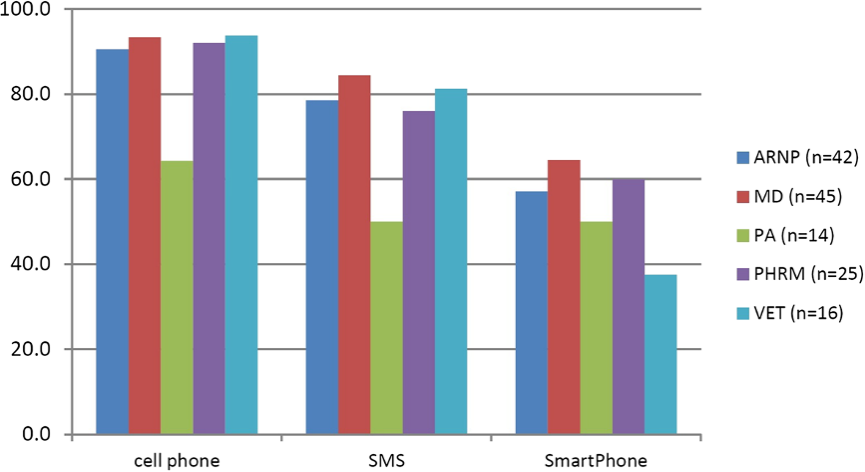
**Health care providers' exposure to receiving professional information by cell phone, SMS, and smart phone (Part 2 providers).**

The technologies through which providers receive professional information and their ownership of SMS-capable devices might impact their ability to shorten a long public health message into a 160 character constrained equivalent. The Part 2 sample reported high rates of receiving information through cell phone, text messaging/SMS and smart phone devices (see Figure 3).

#### Exercise: conversion of public health message to SMS

The exercise presented three messages for conversion to SMS generated by different public health agencies regarding three different topics: a local health jurisdiction message regarding Norovirus; a state health department regarding Rocky Mountain Spotted Fever (RMSF); and a Centers for Disease Control and Prevention (CDC) message regarding Monkeypox. Providers were instructed to feel free to use abbreviations. While there was some variance in the components, every original message included Topic, Recommend, Location, Signs/Sx, Population, Link to More Info, and Source components. No message included the "OtherConditions" component. Table 1 lists the components included in each original full-length message and examples of SMS conversions submitted by providers.

Provider-constructed SMS messages were coded by two experienced qualitative data analysts into each message component group (see Table 4 list). Although the majority of providers (93.7%) completed all three message conversions, because each message varied in content and components we examined each message independently for inclusion of components on a per message basis and association between inclusion of components and exposure to SMS and smart phone ownership.

Over 37% of those who completed the Monkeypox exercise included the source (i.e., CDC) in the converted message, a higher inclusion rate than the other messages (Table 5A.1). The per message analysis identified a high percentage of respondents including a URL in any SMS message, ranging from 61% to over 72% (Table 5A.2).

**Table 5:** **Likelihood of including message source and link to more information (URL) in individual message analysis**

A. Frequency of inclusion:	RMSF	Monkeypox	Norovirus
**1. Was message source included in SMS?**	3/139 (.02%)	51/135 (37.78%)	2/138 (.01%)
**2. Was a URL included in SMS?**	101/139 (72.7%)	93/135 (68.9%)	85/138 (61.6%)
**B. Likelihood of including URL if:**	**RMSF**	**Monkeypox**	**Norovirus**
**1. Receives information by SMS**	3.32 times	1.65 times	2.33 times
	more likely*	more likely	more likely*
**2. Owns a smart phone**	1.48 times	1.40 times	1.33 times
	more likely	more likely	more likely
**3. Owns a smart phone AND receives information by SMS**	1.53 times	3.35 times	1.92 times
	more likely	more likely*	more likely

*statistically significant.

We found statistically a statistically significant association between providers who could receive information by SMS and their inclusion of a link to more information in the RMSF and Norovirus exercises, as compared to providers who reported they could not receive information by SMS (Table 5B.1). Ownership of a smart phone increased the likelihood of including a URL in the message but it was not statistically significant (Table 5B.2). When ownership of a smart phone and receiving information by SMS were combined as an aggregate variable, we found a statistically significant association between both receiving information by SMS and owning a smart phone and including a URL in the Monkeypox exercise, but not in the RMSF and Norovirus exercises (Table 5B.3).

## Discussion

Text messaging could be a valuable public health tool for communicating with health care providers. However, little is known about how to craft these messages for the provider target audience. To our knowledge, this is the first study to investigate the message content and formatting requirements of providers with respect to public health alerts and advisories disseminated by SMS. In addition, rather than asking participants to simply respond to survey questions and speculate on what message components and format presentations are most useful, our inclusion of a participatory exercise asking health care providers to demonstrate their needs is both innovative and especially informative.

In the first part of the survey, it is notable that Source, the component that would provide authority for a message, was one of the least frequently selected components. Yet, in the second part of the survey, over 37% of those who completed the Monkeypox exercise included CDC as the message source, as compared to the other messages. This may indicate that a known authority like CDC will be more likely to be included in a SMS to lend higher credibility. Also notable is that although the conversion messages were generated by public health agencies, information regarding how to contact or submit reports to public health were not selected as important components of a message.

Including a URL is a formatting decision that can improve the effectiveness of a SMS message and expand its content beyond the 160 character limitation. Results regarding including a URL are especially valuable: although only 53.6% of respondents indicated inclusion of a link is essential in a public health message, the proportion increased to 61.6%-72.7% when health care providers converted messages and presumably sought to include as much information as possible within the 160 character constrained message. This shift in including a URL is a finding that would not have been uncovered had the study methods not included the participatory exercise. Further exploration of this finding, using active, participatory methods, is needed not only with health care providers but other public health stakeholder groups.

The statistically significant association between providers who could receive information by SMS and including a URL, as compared to providers who reported they could not receive information by SMS, may be attributed to provider exposure to SMS. It would be likely that health care providers who send or receive text messages would be more familiar with, and thus more likely to, include a URL in a message. As stated previously, the use of mobile technologies, including SMS, are increasing in health care settings. SMS messages are delivering appointment and medication adherence reminders, chronic disease management messages, physician decision support, among other applications and interventions [9, 10]. As SMS increasingly penetrates the clinical environment, its familiarity and acceptability may increase in parallel, along with expectations that public health communications, especially urgent messages, be delivered through mobile devices.

Further exploration of how to effectively use SMS and other mobile technologies to deliver time-sensitive public health information to health care providers, and other public health audiences, is needed, particularly for public health emergency and preparedness response and recovery efforts. Effective public health communications play a central role in minimizing negative outcomes of an emergency, disaster or crisis situation and protecting public safety and welfare [11]. Analyses of events such as 9/11, the 2001 anthrax attacks, threat of pandemic influenza, extreme weather events such as ice storms, hurricanes and tornados, shootings, and botulism outbreaks, among numerous other examples, consistently reveal gaps in effective communications [12, 13]. As a communication modality, SMS is more reliable and stable in an emergency as compared to voice transmission [14] and thus warrants further investigation as a front-line public health tool. It is known that relevant components of emergency communication include message content; communication channel; mechanism of communication which can include the delivery device; quantitative aspects of the communication such as timeliness, frequency and duration of messages; and the target audiences. In addition, more qualitative components of emergency communication include trust in the source and content of the message; impact of the communications on improving knowledge, supporting decision-making, and changing behavior; and relevance of the information exchange for the target audience, among others [15]. If SMS is to be part of public health's toolkit, public health needs more evidence to inform how to craft and deliver messages in this abbreviated format.

### Limitations

This study has several limitations. First, subjects were recruited from a completed randomized controlled trial that examined traditional and mobile messaging. In this study health care providers were randomized to receive quarterly, time-sensitive public health messages via email, fax, SMS or to a control group that did not receive messages for 9–12 months [1]. It is unknown which exposure group subjects had been assigned to in the parent study nor is it possible to know whether exposure in the parent study may have impacted responses in this sub-study. In addition, by recruiting subjects from providers who had completed their participation in the parent study, we may have introduced a selection bias that is not possible to identify or account for in our results. Another limitation is that our sample size was small and some stratifying demographic variables (provider type, gender, etc.) were missing which may have impacted our analyses.

## Conclusions

This work explored how to effectively utilize these technologies to maximize message content, source trust, relevance and motivation to access further information regarding a public health event. A substantive recommendation derived from this study is that public health agencies include a link to additional website information when sending messages in SMS format. While further investigation with respect to crafting SMS messages by health care providers and other key public health audiences is needed to inform use of this technology for public health communications, our study demonstrates that any future research needs to include active participation to ensure results are useful and informative.
